# Cystic Dystrophy of the Duodenal Wall with Underlying Heterotopic Pancreas

**DOI:** 10.5334/jbsr.2860

**Published:** 2022-09-20

**Authors:** Olaia Chalh, Khaoula Sibbou, Ittimad Nassar

**Affiliations:** 1 Ibn Sina Teaching Hospital, MA

**Keywords:** cystic dystrophy, duodenal wall, heterotopic pancreas, computed tomography, MRCP

## Abstract

**Teaching Point:** Cystic dystrophy of the duodenal wall is often associated with underlying heterotopic pancreas and easily assessed with advanced and noninvasive imaging modalities such as CT scan and MRCP.

## Case History

A 56-year-old man presented with recurrent epigastric pain, projectile vomiting, and weight loss. He had a history of chronic alcohol abuse and acute necrotizing pancreatitis one year prior.

Physical examination revealed a tenderness with sensation of induration in the epigastric region.

Laboratory tests showed high level of lipase 870 U/L; (870 UI/L) other results were unremarkable.

**Figure 1:**
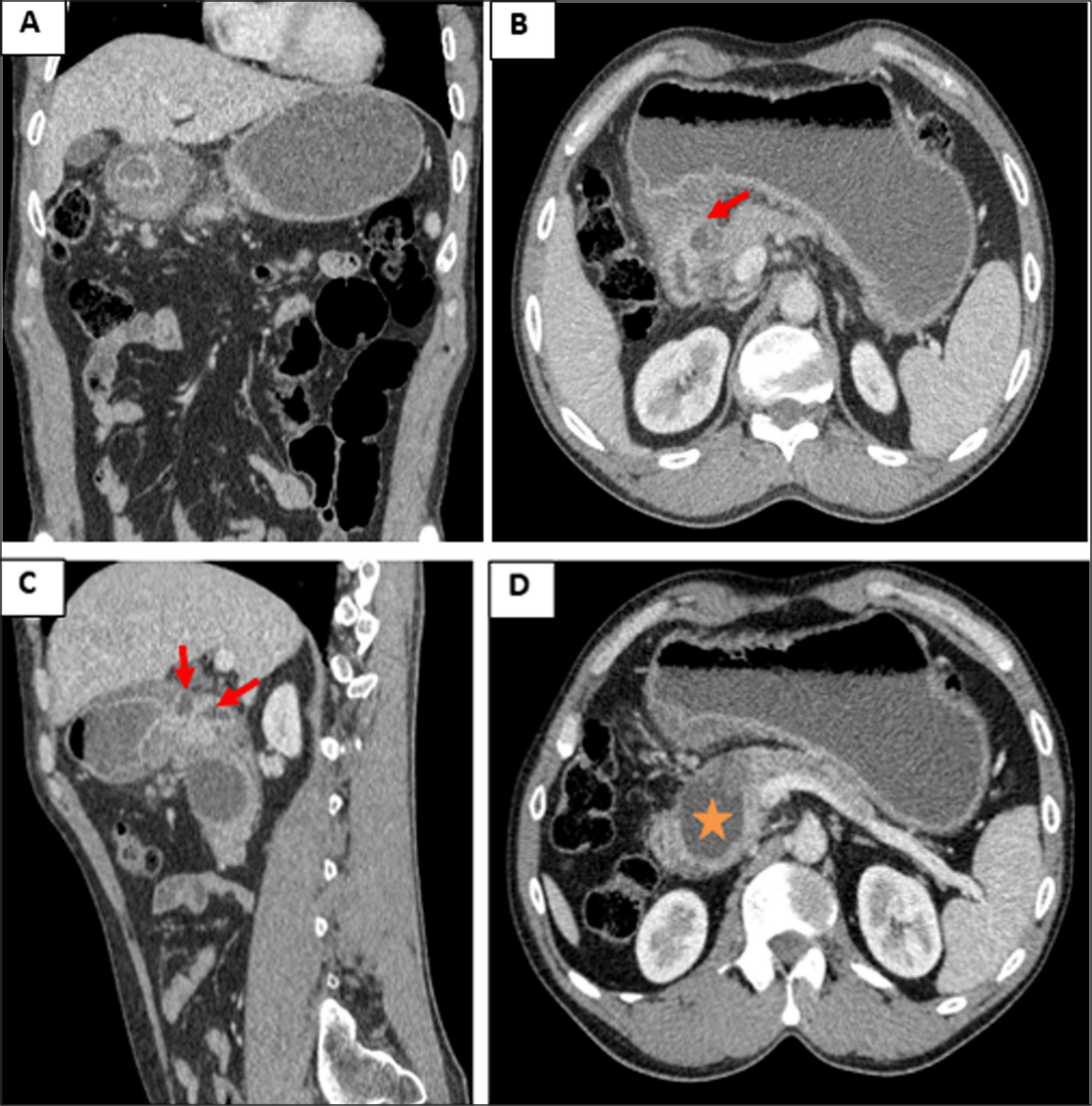


**Figure 2:**
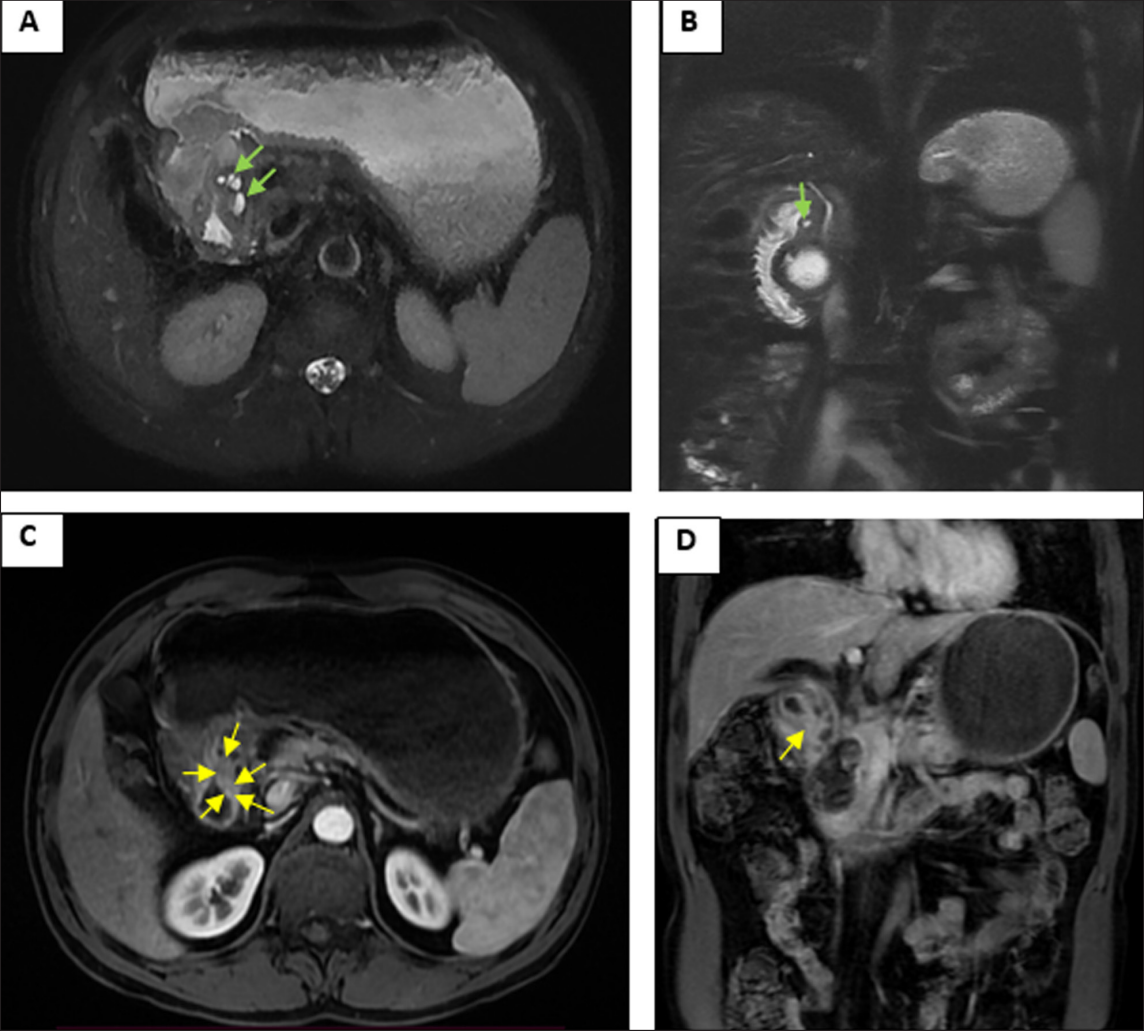


Contrast-enhanced computed tomography (CT) scan (Figure 1A–D) showed a distended stomach and circumferential thickening of duodenal bulb wall with small intramural cysts (red arrows) and narrowing of duodenal lumen. A thick-walled fluid collection in the pancreatic head containing internal debris (asterisk) was also noticed. There were no calcifications of pancreatic parenchyma. Magnetic resonance cholangio-pancreatography (MRCP) confirmed the presence of intramural cysts as small foci of high signal intensity in T2 single shot fast spine echo (green arrows), with adjacent small deposits (yellow arrows) having similar features as normal pancreatic parenchyma in morphological and hemodynamic sequences (Figure 2A–D). Diagnosis of cystic dystrophy of the duodenal wall (CDDW) with underlying heterotopic pancreas was suggested and confirmed by pathological examination after Whipple procedure.

## Comment

CDDW is a rare condition characterized by the presence of cysts within thickened duodenal wall. It often reflects cystic and inflammatory changes of aberrant pancreatic tissue into the duodenal wall, also referred to as pancreatic heterotopia [1]. CDDW occurs most commonly in the second portion of duodenum. It is predominantly seen in males aged between 40 and 50 years old with a history of chronic alcohol abuse. Clinical symptoms include epigastric pain, vomiting, obstructive jaundice, and weight loss. CT scan, MRCP, and endoscopic retrograde cholangio-pancreatography (ERCP) are the main imaging modalities for the diagnosis [1].

Contrast-enhanced CT scan and magnetic resonance imaging (MRI) are helpful for this challenging diagnosis. Demonstration of mural thickening, stenosis of duodenal lumen, and cysts within the duodenal wall or in the paraduodenal groove are the key features. Small remnants of pancreatic tissue within duodenal wall having the same morphological and hemodynamic behavior as normal pancreas parenchyma may be detected. MRCP is superior in demonstrating dilatation of small pancreatic ducts into pancreatic remnants [1].

ERCP remains the modality of choice in confounding cases. It may confirm the intramural location of the cysts and demonstrate signs of chronic pancreatitis. Using a fine-needle aspiration, ERCP may rule out duodenal adenocarcinoma, which is the main differential diagnosis of CDDW. Groove pancreatitis still the main complication of CDDW. in such cases, conservative treatment is recommended. However, surgical options such as pylorus-preserving pancreatectomy and the Whipple procedure are necessary to alleviate recurrent symptoms [1].
